# Flexor Hallucis Longus Transfer And V-Y Plasty: An Effective Treatment Modality for Chronic Achilles Rupture - A Case Series

**DOI:** 10.5704/MOJ.2311.009

**Published:** 2023-11

**Authors:** RH Rashid, R Ali, M Zahid, M Ali, T Ahmad

**Affiliations:** Section of Orthopaedics, Aga Khan University Hospital, Karachi, Pakistan

**Keywords:** flexor hallucis longus transfer, chronic Achilles tendon rupture, insertional Achilles tendinopathy

## Abstract

**Introduction:**

To assess outcomes of FHL transfer and V-Y plasty for chronic Achilles rupture due to insertional Achilles tendinopathy.

**Materials and methods:**

A case series of 12 patients was conducted between 1st January 2017 and 31st December 2018. The patients had short flexor hallucis longus tendon transfer with gastrocnemius lengthening by V-Y plasty for Achilles tendon rupture. Patients were allowed full weight bearing at six weeks post-operatively, and were followed up at three months and six months post-operatively, when the range of motion of the ankle was examined, and the outcome was assessed using the EFAS score.

**Results:**

Of the 12 patients in the study, the majority were males; the mean age was 50.6±8.96 years. A significant improvement in dorsiflexion and plantarflexion was noted at the six-month follow-up compared to the three-month follow-up (P=<0.001 for both). When compared to the normal side, dorsiflexion and plantarflexion of the affected ankle were significantly less at three months but were comparable at six months post-operatively. A significant improvement was noted in the mean EFAS score at the six-month follow-up (25.5±5.71) compared to three months (18.6±0.90) post-surgery (P=0.001). Males were also noted to have significantly higher EFAS scores at their six-month follow-up than females (P=0.022). In contrast, a negative correlation was noted between the European Foot and Ankle Society (EFAS) score at the final follow-up and age (P=0.011).

**Conclusion:**

FHL tendon transfer with V-Y plasty in chronic Achilles rupture due to insertional Achilles tendinopathy is an effective procedure resulting in the restoration of the ankle range of motion and improvement in functional scores.

## Introduction

Achilles tendon rupture is one of the most commonly encountered tendon injuries caused by trauma or conditions such as insertional Achilles tendinopathy, Achilles tendinosis, Haglund deformity, or retrocalcaneal bursitis^1^. Amongst these, insertional Achilles tendinopathy is a degenerative disorder which causes considerable heel pain leading to gait abnormalities, with limitation of routine activities^1,2^. Appropriate management of this condition may prevent Achilles tendon rupture; however, patients often present with a ruptured tendon following months or years of pain^3^.

Achilles tendon rupture is mainly a clinical diagnosis. Despite careful clinical examination, 10% - 25% of ruptures can be missed^3,4^. When the condition is left untreated for more than four to six weeks, it is considered a chronic rupture^5,6^. Management of these chronic ruptures often requires operative management, with the main aim of surgery being the alleviation of pain and restoration of functionality^7,8^.

Various surgical options for treating chronic Achilles tendon rupture have been reported in the literature. V-Y plasty, gastrocnemius-soleus turndown flap, and local tendon transfers, including flexor hallucis longus (FHL), are some of the commonly practiced techniques. Other procedures include the use of synthetic ligaments^5,6^. Although multiple procedures are available for the treatment of chronic Achilles tendon rupture, there is still a lack of evidence supporting one technique over another.

FHL tendon transfer for chronic Achilles rupture was first described in 1993 by Wapner *et al* ^9^. FHL tendon has higher durability and more strength than the other nearby tendons, thereby making it a more feasible option for transfer. Previous studies worldwide have also shown good patient satisfaction and functional outcomes with FHL tendon transfer for Achilles tendon rupture^7,10,11^. However, to the best of our knowledge, no international literature on FHL tendon transfer for chronic Achilles tendon rupture from the Asia-Pacific region is available. Hence, the aim of this study was to assess outcomes of FHL transfer for chronic Achilles tendon rupture due to insertional Achilles tendinopathy from this part of the world.

## Materials and Methods

A single-centre prospective case series was conducted between 1st January 2017 and 31st December 2018 at a tertiary care teaching centre in Pakistan. All patients who underwent FHL transfer for chronic Achilles tendon rupture due to insertional Achilles tendinopathy within the mentioned duration were included, after a trial of conservative treatment for up to three months, and non-probability consecutive sampling was performed. Diagnosis of chronic Achilles tendon rupture was made on the presence of Haglund deformity and calcaneal exostosis on radiographs. Skeletally immature individuals (aged <18 years) and individuals with any other skeletal deformities or those diagnosed with congenital syndromes were excluded from the study. Exemption from the Ethical Review Committee (ERC) at our institution was obtained prior to the conduction of the study (ERC No: 2020-5324-14083).

**Fig 1:**
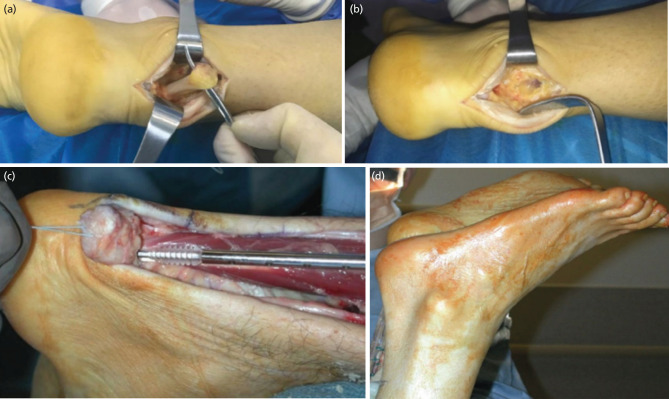
(a) Identification of diseased portion of Achilles tendon. (b) Achilles’ tendon defect after excising diseased portion. (c) Tenodesis of Flexor Hallucis Longus tendon. The image represents insertion of the interference screw holding the Flexor Hallucis Longus tendon into the bone tunnel. Reprinted from Operative Techniques in Foot and Ankle Surgery (2nd ed., p.1122) by Easley ME and Wiese SW, 2017, Wolters Kluwer. Copyright 2017 by Wolters Kluwer. (d) Resting tension of Achilles tendon after repair. The image represents comparison of the resting Achilles tendon tension between the repaired (right) and the normal (left) side, which should be equal. Reprinted from Operative Techniques in Foot and Ankle Surgery (2nd ed., p.1122) by Easley ME and Wiese SW, 2017, Wolters Kluwer. Copyright 2017 by Wolters Kluwer.

Baseline demographic data, including age, gender, co-morbidities, and site of injury, was recorded for all patients. All surgeries were performed under general anaesthesia by a fellowship-trained foot and ankle surgeon. All patients were positioned in a prone position, and a tourniquet was applied. After standard preparation and draping, a posteromedial longitudinal incision was made to prevent wound complications. The Achilles tendon was identified, the diseased portion was thoroughly debrided, and the gap was measured (Fig. 1a and b). Next, the deep posterior compartment of the leg was dissected to expose the belly and tendon of FHL.

The tendon of the FHL was divided as distally as possible by keeping the ankle and big toe in maximum plantar flexion. In order to prevent damage to the neurovascular structures, the direction of dissection was kept from medial to lateral. The tendon was then sutured with Krakow stitches and passed through the tendon sizer for adequate bone tunnel preparation.

A guide wire was then passed into the calcaneum, just anterior to the Achilles tendon insertion site. Reaming was done over the guide wire in accordance with the FHL tendon diameter. Care was taken not to penetrate the plantar surface of the calcaneum while reaming. With the help of a beath needle, the ends of the suture holding the FHL tendon were then passed into the bone tunnel. The guide wire was pulled through the plantar surface of the calcaneum to place the FHL tendon inside the tunnel, which was reamed, following which tenodesis was done using an interference screw of appropriate size (Fig. 1c). Tension on the tendon was maintained by applying traction to the suture. The ideal tension at which the FHL should be inserted is one that keeps the ankle at a resting tension equal to that of the contralateral side.

After the FHL transfer was completed, tension was given to the Achilles tendon. A proximal V-Y plasty was done to restore the length of the Achilles tendon. V-Y plasty can adequately cover a gap of about 5cm. A 4.75mm suture anchor was inserted into the calcaneum, and the Achilles tendon was attached. At this moment, the ankle resting tension should be equal to the contralateral side to avoid overtightening (Fig. 1d). The paratenon was then repaired, and the skin was closed in layers. A well-padded anterior splint with resting ankle tension equal to the contralateral ankle was applied^12^.

**Fig 2:**
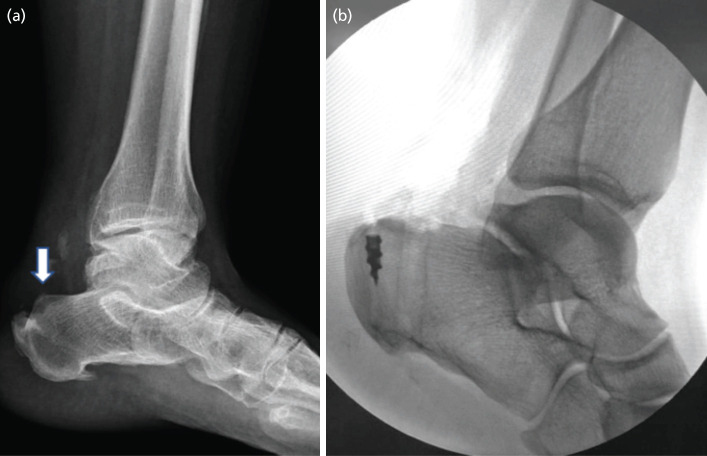
(a) Pre-operative lateral ankle view showing Haglund deformity (white arrow). (b) Post-operative lateral ankle view.

Post-procedure, an anterior below-knee back slab was applied, keeping the ankle in a neutral position for six weeks. Non-weight-bearing ambulation was allowed, and all patients were initially followed up in the clinic for the removal of stitches after two weeks. Patients were allowed full weight bearing after six weeks post-operatively, and physiotherapy with eccentric exercises to mobilise the ankle and strengthen the Achilles tendon was started. Pre- and post-operative radiographs are shown (Fig. 2).

Our primary objective was to assess the functional outcome after the procedure using the European Foot and Ankle Society (EFAS) score at three- and six-month postoperatively^13^. This score included ten questions related to daily and sports activities, graded on a scale of 0-4, with 0 representing impossible to perform while 4 representing able to perform with no limitations. A higher score represented a better functional status. An assessment of the range of ankle motion was also performed at three- and six-month follow-up as part of our secondary objective.

Data was analysed using SPSS version 21. All qualitative variables were expressed as percentages, while quantitative variables were represented as mean±standard deviation. Independent samples and paired T-tests were used where appropriate to identify any associations and one-way ANOVA was used to compare two means when required. A P-value of <0.05 was considered to be significant.

## Results

In total, 12 patients were enrolled in the study. The majority of the patients were males, and there was equal distribution in terms of the site of the injury, as shown in Table I. The mean age of the study population was 50.6±8.96 years (range: 30 - 63 years). The mean defect size of the Achilles tendon was noted to be 7.38±1.0cm.

**Table I:** Demographic characteristics of the study population.

Characteristics	No. of participants (%)
Gender	
Male	8 (66.7)
Female	4 (33.3)
Co-morbidities	
Diabetes Mellitus	4 (33.3)
Hypertension	6 (50.0)
Smoking Status	
Smoker	5 (41.7)
Non-smoker	7 (58.3)
Site of Injury	
Right	6 (50.0)
Left	6 (50.0)

With regards to the functional outcome, the mean EFAS score at three months post-surgery was 18.6±0.90, significantly improving to 25.5±5.71 at the six-month follow-up (P=0.001), as represented in Table II. Males were also noted to have significantly higher EFAS scores at their six-month follow-up than females (P=0.022). A negative correlation was also noted between EFAS scores at the final follow-up and age (P=0.011), representing higher EFAS scores for younger individuals.

**Table II:** Comparison of EFAS scores.

Independent Variables	EFAS Score (mean ± S.D*)	P-value
Follow-up Duration		0.001
three months	18.58 ± 0.90	
six months	25.5 ± 5.71	
Gender (three months follow-up)		0.67
Male	18.5 ± 0.93	
Female	18.75 ± 0.96	
Gender (six months follow-up)		0.022
Male	27.6 ± 5.81	
Female	21.3 ± 2.22	

*Standard Deviation

**Fig 3:**
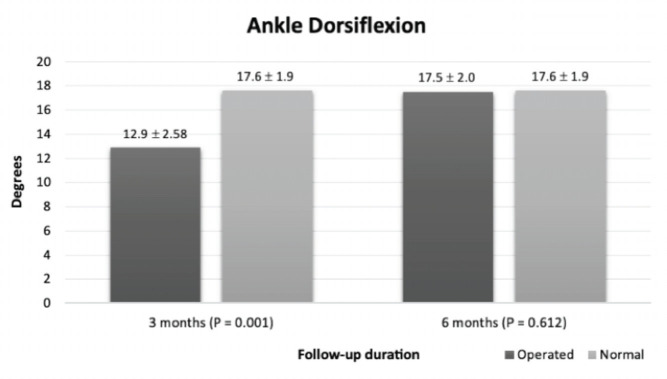
Comparison of ankle dorsiflexion between operated and normal side.

**Fig 4:**
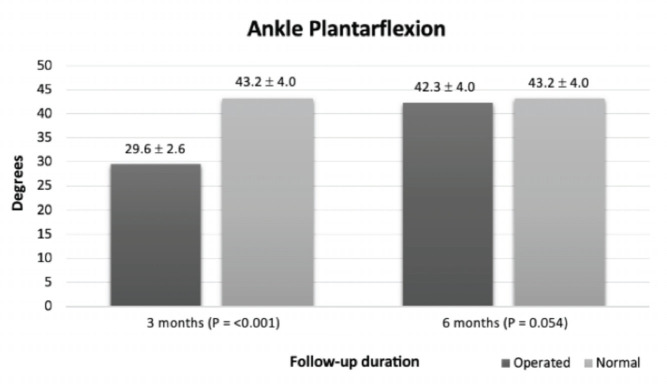
Comparison of ankle plantarflexion between operated and normal side.

Table III shows the mean post-operative range of motion at three-month and six-month follow-ups. A significant improvement in dorsiflexion and plantarflexion was noted at a six-month follow-up compared to three months postoperatively (P=<0.001 for both). When compared to the normal side, the dorsiflexion and plantarflexion of the affected ankle were significantly lower at the three-month follow-up, with a P-value of <0.001 for both, as shown in Fig. 3 and Fig. 4, respectively. However, at the six-month follow-up, dorsiflexion and plantarflexion at the operated ankle were comparable to the unaffected side (P=0.612 and 0.054, respectively).

**Table III:** Comparison of ankle range of motion between three and six months follow-up.

Ankle Range of Motion (mean ± S.D*)	Follow-up duration		P-value
	Three months	Six months	
Dorsiflexion	12.9 ± 2.58°	17.5 ± 1.98°	0.001
Plantarflexion	29.6 ± 2.58°	42.3 ± 3.96°	<0.001

*Standard Deviation

No significant correlation was noted between the length of the Achilles tendon defect and outcome variables, including EFAS scores and range of motion at three and six months. Smoking status, presence of co-morbidities and side of surgery also had no significant effect on the outcomes.

## Discussion

Achilles tendon rupture has a significant effect on gait and can result in a calcaneal limp. Loss of plantar flexion of the ankle joint also leads to ankle instability, loss of control of forward rotation of the tibia during the stance phase, and excessive vertical movement of the centre of gravity of the body^13^. Therefore, effective treatment of the Achilles tendon rupture is important to restore normal gait and hence improve the overall functional status and quality of life.

Surgical treatment for chronic Achilles tendon rupture is indicated if conservative measures fail to alleviate the symptoms, and various surgical modalities have been used in the treatment^1^. V-Y advancement flap is one of the commonly used procedures, which was first described in 1975 by Abraham and Pankovich^14,15^. Other authors have also favoured the use of the Achilles tendon turndown flaps for >5cm defects^16,17^. However, with regard to these procedures, there have been concerns related to the requirement for large incisions and their effect on the muscle length-tension relationship^14^.

To address these issues, local tendon transfers have gained considerable popularity in recent times. There have been various studies that have identified peroneus brevis (PB), flexor digitorum longus (FDL), and FHL as potential options for tendon transfers^18,19^. The current study reports significantly improved outcomes, both in terms of functional status and range of motion, after FHL transfer and V-Y plasty. A significant improvement in EFAS scores was noted six months post-operatively, indicating improved functionality for all patients. Similarly, dorsiflexion and plantarflexion at the ankle joint were also noted to improve significantly at six months compared to that at three months and were found to be equal to that of the contralateral side. These findings are a testament to the effectiveness of the procedure at our institute.

Multiple studies have also shown considerable improvement in patient functionality after FHL tendon transfers, with reported AOFAS hindfoot-ankle scores between 91 and 98.3 post-operatively in international literature^10,20^. Den Hartog BD, in his study, showed significant improvement in the American Orthopaedic Foot and Ankle Society (AOFAS) score from 41.7 to 90.1 after FHL tendon transfer to treat chronic Achilles tendinosis. He also noted that the majority of the patients recorded their maximum scores after six months of recovery, with a mean time to maximum improvement being 8.2 months^16^. These results further substantiate the results of the current study, where a significant improvement in the EFAS scores was noted at the six months post-operative interval compared to three months post-operatively.

Transfer of FHL is currently the most preferred procedure for chronic Achilles tendon rupture by orthopaedic surgeons worldwide^14^. Wapner *et al* identified several benefits of FHL transfer over other tendons, including its superior strength compared to other nearby tendons, a similar axis of pull and close anatomical proximity to the Achilles tendon, and the fact that it contracts in the same phase as the Achilles tendon^9^. In a study conducted in Egypt, El-Tantawy and Azzam noted a mean dorsiflexion of 23.2±2.71° and a mean plantarflexion of 39.5±3.47° after FHL transfer. This range of motion was also comparable to the non-operated side^11^. The dorsiflexion and plantarflexion angles in our study at six-month follow-up are also comparable to the reported literature.

Loss of big toe plantar flexion is one of the potential complications of FHL tendon transfer which has been studied in international literature^10^. Although the weakness of plantar flexion at the interphalangeal joint of the big toe has been reported, multiple studies have concluded that this is only a laboratory finding with little or no consequences in the overall functionality^10,21^. The authors of the current study also did not find any clinical evidence of decreased big toe function in any of the patients.

Similar to the results of the present study, Abubeih *et al* in 2018 also concluded that younger patients had significantly greater improvement in their outcome scores compared to older individuals^12^. Young individuals are likely to be more active and participate more in physical activities than the elderly. This could explain the better outcomes in this age group, as increased activity can lead to an early adjustment to the changed biomechanics following a tendon transfer procedure.

This study reports our experience and outcome of FHL transfer performed in 12 cases of Achilles rupture due to insertional tendinitis and provides data to support the use of FHL transfer for chronic Achilles tendon rupture, especially in the Asia-Pacific, which is still a developing region with a lack of resources and facilities. However, the small sample size and lack of a control group are limitations that can be addressed in future studies, along with an increased follow-up duration, to provide definite conclusions. To the best of our knowledge, this is the first study highlighting the outcomes of this particular procedure from our region, and we hope that this paves the way for further large-scale analytical studies from this part of the world.

## Conclusion

FHL tendon transfer with V-Y plasty in chronic Achilles tendon rupture due to insertional Achilles tendinopathy is an effective procedure resulting in restoration of the range of motion and improved functional scores of the ankle. However, further multi-centre analytical studies are recommended for definite conclusions.
